# The Response of *Pseudomonas aeruginosa* PAO1 to UV-activated Titanium Dioxide/Silica Nanotubes

**DOI:** 10.3390/ijms21207748

**Published:** 2020-10-20

**Authors:** Adrian Augustyniak, Krzysztof Cendrowski, Bartłomiej Grygorcewicz, Joanna Jabłońska, Paweł Nawrotek, Martyna Trukawka, Ewa Mijowska, Magdalena Popowska

**Affiliations:** 1 Department of Chemical and Process Engineering, Faculty of Chemical Technology and Engineering, West Pomeranian University of Technology, Szczecin, Piastów Avenue 42, 71-065 Szczecin, Poland; joanna_jablonska@zut.edu.pl; 2 Chair of Building Materials and Construction Chemistry, Technische Universität Berlin, Gustav-Meyer-Allee 25, 13355 Berlin, Germany; 3 Department of Microbiology and Biotechnology, Faculty of Biotechnology and Animal Husbandry, West Pomeranian University of Technology, Szczecin, Piastów Avenue 45, 70-311 Szczecin, Poland; pawel.nawrotek@zut.edu.pl; 4 Department of Nanomaterials Physicochemistry, West Pomeranian University of Technology, Szczecin, Piastów Avenue 45, 70-311 Szczecin, Poland; krzysztof.cendrowski@zut.edu.pl (K.C.); martyna.barylak@zut.edu.pl (M.T.); ewa.borowiak-palen@zut.edu.pl (E.M.); 5 Chair of Microbiology, Immunology and Laboratory Medicine, Department of Laboratory Medicine, Pomeranian Medical University in Szczecin, Powstańców Wielkopolskich Avenue 72, 70-111 Szczecin, Poland bartlomiej.grygorcewicz@pum.edu.pl; 6 Department of Bacterial Physiology, Institute of Microbiology, Faculty of Biology, University of Warsaw, Warsaw, Miecznikowa Street 1, 02-096 Warsaw, Poland; magdapop@biol.uw.edu.pl

**Keywords:** bacterial physiology, cells agglomeration, stimulation, silica nanocomposite

## Abstract

*Pseudomonas aeruginosa* is a bacterium of high clinical and biotechnological importance thanks to its high adaptability to environmental conditions. The increasing incidence of antibiotic-resistant strains has created a need for alternative methods to increase the chance of recovery in infected patients. Various nanomaterials have the potential to be used for this purpose. Therefore, we aimed to study the physiological response of *P. aeruginosa* PAO1 to titanium dioxide/silica nanotubes. The results suggest that UV light-irradiated nanomaterial triggers strong agglomeration in the studied bacteria that was confirmed by microscopy, spectrophotometry, and flow cytometry. The effect was diminished when the nanomaterial was applied without initial irradiation, with UV light indicating that the creation of reactive oxygen species could play a role in this phenomenon. The nanocomposite also affected biofilm formation ability. Even though the biomass of biofilms was comparable, the viability of cells in biofilms was upregulated in 48-hour biofilms. Furthermore, from six selected genes, the *mexA* coding efflux pump was upregulated, which could be associated with an interaction with TiO_2_. The results show that titanium dioxide/silica nanotubes may alter the physiological and metabolic functions of *P. aeruginosa* PAO1.

## 1. Introduction

*Pseudomonas aeruginosa* is one of the major etiological agents that causes nosocomial infections [1,2]. Patients suffering from diabetes, cystic fibrosis, or burn wounds are the most susceptible to infections caused by pseudomonads. The number of urinary tract infections caused by this Gram-negative, rod-shaped, and motile bacterium has increased in recent years, where it was isolated from 7–15% of cases in exchange with *Klebsiella pneumoniae*. Antibiotic-resistant pseudomonads constituted over 13% of isolates [3,4,5]. Pseudomonads can produce a variety of virulence factors including type IV adhesive fimbriae, exotoxin A (a member of the family mono-ADP-ribosyltransferases), enzyme S (inhibiting lipid synthesis), and phospholipase C, that together with rhamnolipids disrupt membrane phospholipids. Furthermore, they can produce an alginate sheath on the cells’ surface [2,3,6]. One of the main problems associated with infections caused by pseudomonads is their ability to quickly develop resistance to clinically used antibiotics [7]. For example, multiple efflux pumps facilitate higher resistance in pseudomonads [8]. This issue was highlighted in the World Health Organization statement released in February 2017 in which carbapenem-resistant *P. aeruginosa* was acclaimed as number 2 on the list presenting the 12 most hazardous bacteria in healthcare [9].

Nanomaterials are used in almost every aspect of life, including water purification, agriculture, and novel building materials [10,11,12,13]. Starting with nanoparticles, researchers have developed methods for the synthesis of more advanced nanomaterials expressing various shapes (e.g., triangular, cubic, pyramidal, etc.) and sizes (e.g., quantum dots or nanoparticles) [14]. A material in which at least one phase is in the nanoscale can form a nanocomposite such as titanium dioxide/silica nanotubes, where mesoporous silica in tubular form is functionalized with titanium dioxide [15]. The functionalization of nanomaterials aims to produce composite nanomaterials that act differently than the structures from which they are composed [16,17].

Nanostructures have often been proposed for antimicrobials as well as suggested for models to study them [18,19,20]. There are many data on the effects of nanoparticles on microorganisms [21]. On the other hand, studies on more complex nanomaterials (nanocomposites) are still in the minority. The combination of nanomaterials can change the properties of the product nanostructure [10,16,17]. For that reason, it is necessary to investigate the applicability of nanomaterials in various branches of science and technology. While many studies regarding nanomaterials are focused on the whole bacterial consortia, one should note that this approach may not offer answers on the specific interactions which can lead to discoveries concerning the biotechnological use of microorganisms stimulated by nanomaterials [22,23]. Although understanding the reactions of the whole microbial community is advantageous and helpful in predictive investigations, knowledge about specific reactions may provide data that allow the control of phenomena observed during the interaction between nanostructures and bacteria or fungi, enabling them to be used with certain advantages (e.g., biotechnological purposes) [24,25].

Pseudomonads (including *P. aeruginosa*) are known for their high biotechnological potential. Thanks to their high adaptability, these microorganisms were proposed for use in the bioremediation of contaminated sites [26]. Moreover, bacteriocins (pyocins) produced by *P. aeruginosa* have the potential to be used against bacteria resistant to antibiotics [27,28]. These bacteria may also exhibit extraordinary reactions to nanomaterials. Similar to resistance to drugs, they can show higher resistance to nanomaterials, including quantum dots (QD). It seems that the response to QD triggers global defense mechanisms in cells, which can dissolve, remove, or transform nanoparticles into more biocompatible structures [29]. These bacteria have also shown an ability to disperse TiO_2_ nanoparticles as described by Horst et al. [30]. It is known that bacteria can accumulate ions and form nanoparticles inside cells. Thanks to this phenomenon, *Pseudomonas* spp. have been used to synthesize nanoparticles, e.g., gold and silver nanoparticles [31,32]. Furthermore, their metabolites can also support the synthesis of stable nanoparticles. Surfactants (rhamnolipids) isolated from *P. aeruginosa* were used by Farias et al. [33] for the synthesis of silver nanoparticles. Therefore, studying the response of this bacterium to novel nanomaterials may uncover new phenomena that could be useful in biotechnology and medicine.

In this study, we investigated selected physiological characteristics of *P. aeruginosa* in response to UV-activated titanium dioxide/silica nanotubes.

## 2. Results

### 2.1. Physical and Chemical Characterization of Studied Nanomaterial

**Figure 1:**
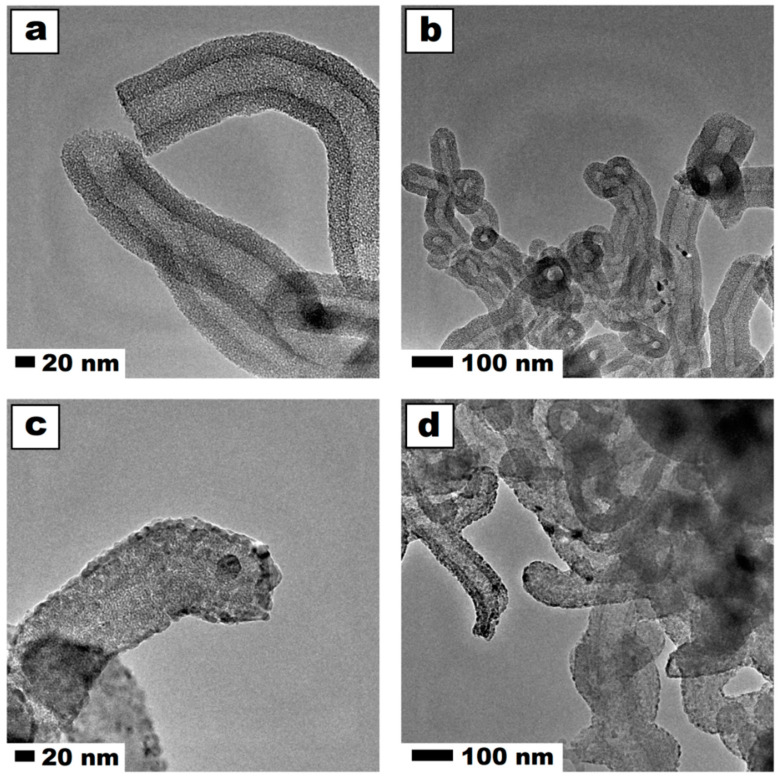
TEM images of pristine (**a**,**b**) and titanium dioxide functionalized (**c**,**d**) mesoporous silica nanotubes.

The detailed physical structure and chemical composition of the mesoporous silica nanospheres functionalized with titanium dioxide were previously described by Cendrowski et al. [15,34]. In these studies, high-resolution transmission electron microscopy (TEM) images and energy-dispersive X-ray spectroscopy (EDS) maps of elemental distribution proved the successful coating of mesoporous silica and the introduction of titanium dioxide to the mesopores. The additional verification confirmed the morphological modification based on the surface analysis through the N_2_ adsorption isotherm. Carbon nanotubes coated with mesoporous silica had an enhanced surface area. Further functionalization with titanium dioxide decreased their surface area by a factor of six, due to the filling of the channels by titanium dioxide. Furthermore, the removal of carbon nanotubes increased the specific surface area of this sample to 463 m^2^/g [15]. The representative images of the nanomaterial confirmed the previous findings and are shown in Figure 1. Further TEM and scanning transmission electron microscopy images along with EDS mapping of the intermediates and the final product were previously published by Cendrowski [34]. The images in Figure 1c–d show the silica nanotubes after functionalization with titanium dioxide. From the comparison of the images before (a,b) and after (c,d) functionalization, it is clear that crystal structures of titanium dioxide were successfully deposited in the pores and surface of the nanotubes. The darker tone of the crystal accounted for the higher density than silica nanotubes. This observation is in accordance with the elemental mapping that was previously published [34].

**Figure 2:**
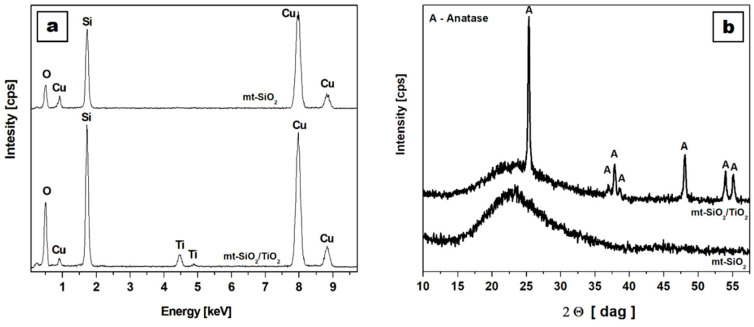
Energy-dispersive X-ray spectra (**a**) and X-ray diffraction pattern (**b**) of pristine (mt-SiO_2_) and titanium dioxide (mt-SiO_2_/TiO_2_) functionalized mesoporous silica nanotubes.

Figure 2a presents the energy-dispersive X-ray spectra of pristine (mt-SiO_2_) and functionalized silica nanotubes (mt-SiO_2_/TiO_2_). EDS analysis confirmed the microscopic observations, showing that mt-SiO_2_ was composed purely of silica, without any contaminations. The obtained peaks accounted for the silicon, oxygen, and copper (coming from the TEM copper grid). After functionalization, an additional peak appeared in the mt-SiO_2_/TiO_2_ material, indicating the presence of titanium in the sample. The X-ray diffraction patterns of the mt-SiO_2_ and mt-SiO_2_/TiO_2_ (Figure 2b) were in accordance with the TEM and EDS analyses. The mt-SiO_2_ pattern showed a broad peak between 15 and 30 degrees, that corresponded to amorphous silica nanomaterials. The mt-SiO_2_/TiO_2_ showed a broad peak from silica and additional peaks that were indexed to the pure tetragonal anatase phase (ICDD #00 021 1272) that was previously described by Cendrowski et al. [15]. In the range of 15 to 30 degrees, the broad peak corresponded to silica in the nanomaterials. The Raman scattering spectra (using a laser beam with an excitation wavelength of 785 nm) of the nanomaterial that were used to confirm the presence of carbon nanotubes at different stages of the synthesis and functionalization were also previously reported and confirmed the complete removal of carbon nanotubes [34].

### 2.2. Viability and Agglomeration

The studied nanomaterial had no distinguishable effect on the morphology of colonies formed by *P. aeruginosa* on solid TSA medium, regardless of the conducted experiment and the concentration of nanomaterial. Thus, no pleomorphic effect was observed on the macroscopic scale. 

**Figure 3:**
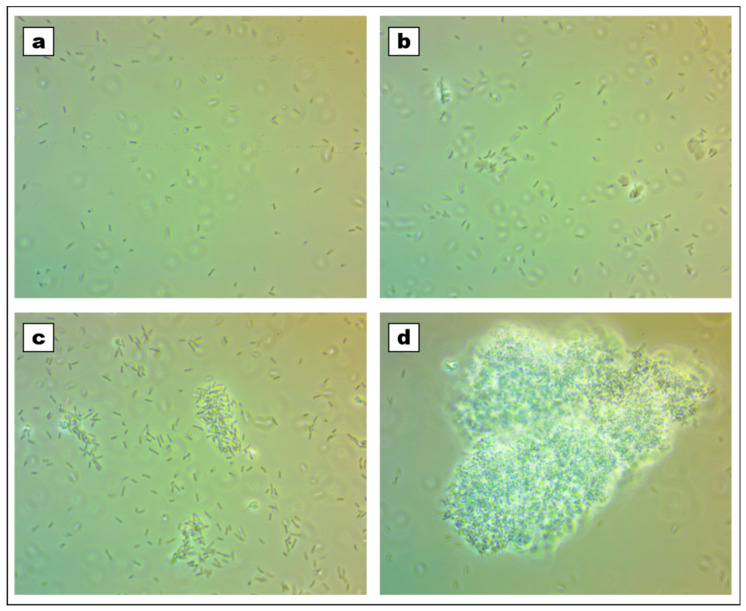
Microphotographs of bacteria in control sample (**a**) and after six hours of incubation with 0.01 mg/mL (**b**), 0.05 mg/mL (**c**), and 0.1 mg/mL (**d**) of titanium dioxide/silica nanotubes; magnification 1000×.

**Figure 4:**
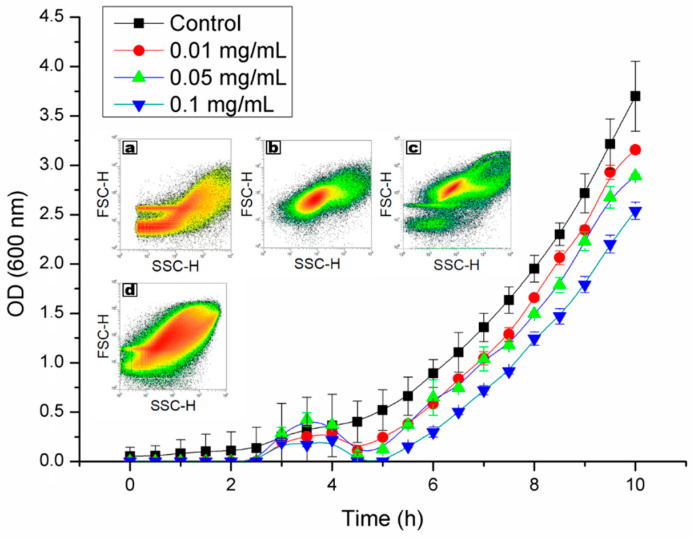
Optical density of *Pseudomonas aeruginosa* incubated with three concentrations of titanium dioxide/silica nanotubes and cytograms results from nanomaterial dispersed in medium with nanomaterial (**a**), *Pseudomonas aeruginosa* PAO1 alone (**b**), and with unexposed (**c**) and UV light-exposed (**d**) titanium dioxide/silica nanotubes.

During the research, an interaction between cells and titanium dioxide/silica nanotubes was established and detected under the microscope. Bacteria cultured in the liquid medium were likely to switch into an agglomeration, which was dependent on the concentration of used nanomaterial (Figure 3). However, this effect was increased when the nanomaterial was irradiated with UV light before the experiment. The microscopic observation was supported by growth kinetics and flow cytometry analysis that also showed the dependence on the concentration of titanium dioxide/silica nanotubes (Figure 4.). Additionally, the alamarBlue^®^ assay performed in the tenth hour of the experiment did not show statistically significant differences between the viability in the cultures. The growth curves were affected in samples containing nanomaterials. According to these results, the peak of agglomeration was between second and fifth hours of culture. This led to a shift in growth time. Similarly, population fingerprinting that was performed by flow cytometry showed an increase in the number of agglomerated objects. This effect was observed only in samples that were irradiated with UV light prior to the contact with bacterial cells (Figure 4a–d). 

### 2.3. Biofilm Formation

**Figure 5:**
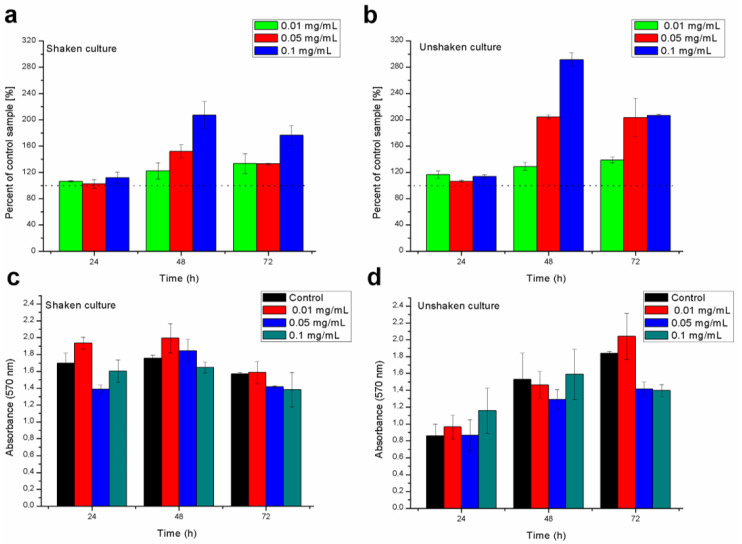
Viability (**a**,**b**) and biomass (**c**,**d**) of biofilms formed by *P. aeruginosa* PAO1 with and without the presence of titanium dioxide/silica nanotubes compared between shaken (**a**,**c**) and unshaken (**b**,**d**) cultures; nanomaterials were used in three concentrations—0.01, 0.05, and 0.1 mg/mL; bars sharing letters are different at *P* < 0.05, letter “a” always indicates a difference with control samples.

The addition of nanomaterials to cultures affected the ability to produce biofilm. The observed reaction was variable depending on the studied conditions (time, shaking). The results from this stage are shown in Figure 5, along with the statistical analysis. Biofilm formation was variable in the scope of different concentrations of the used nanocomposite. The biomass as well as the viability of cells in grown biofilms depended on the time and concentration of titanium dioxide/silica nanotubes. The general trend has shown that even though the biomass of biofilm is not significantly different, there was a considerable increase in the viability, particularly after 48 h of culture. However, it should be underlined that the agitation of samples affected the outcome and the resolution of gained data, particularly in 72-hour-old biofilms.

### 2.4. Relative Expression of Selected Genes 

**Figure 6:**
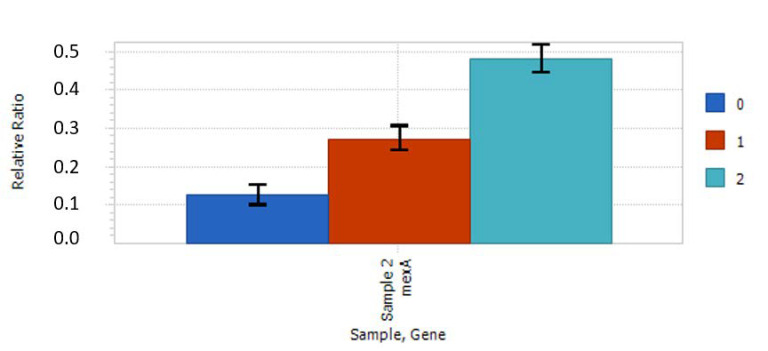
Expression of *mexA* gene (measured in RT-qPCR) in *P. aeruginosa* PAO1 in response to titanium dioxide/silica nanotubes in comparison to the control; 0—0.01 mg/mL, 1—0.05 mg/mL, 2—0.1 mg/mL.

Studies indicated only one significant difference in the expression of studied genes. Bacteria stimulated with the nanocomposite slightly increased (0.5-fold) the expression of the *mexA* gene, which codes an efflux pump. The expression increased with concentration as shown in Figure 6. No other statistically significant relations were noticed. However slight, the tendencies gained in the results were repeatable between experiments.

### 2.5. Antibiotic Susceptibility on Media Containing Activated Nanomaterials

The results show no differences in the size of the inhibition zone diameters between control and tested samples that may be interpreted as the change in susceptibility to the antibiotics. Even though the expression of *mexA* gene coding the efflux pump was slightly increased, the phenotypic test did not confirm that, in the studied condition, *P. aeruginosa* PAO1 was either more or less susceptible to selected antibiotics. Summarized data from this experiment are shown in Table 1.

## 3. Discussion

The agglomeration of cells was confirmed with the use of all selected methods including phase-contrast microscopy, optical density (OD) measurements, and flow cytometry. Additional controls that were not irradiated before studies proved that the use of UV light was the main trigger that induced the agglomeration of cells (Figure 4a–d). The literature data indicate that such irradiation can induce a photocatalytic reaction in TiO_2_ that was deposited in the mesopores of silica nanotubes. In that case, the irradiated nanomaterial releases radicals that can cause oxidative stress to the microorganisms by the generation of superoxide, hydroxyl, and singlet oxygen radicals [35,36]. This explains the reduced agglomeration in samples that were not irradiated and suggests that cells were agglomerating in response to oxidative stress. However, silica probably served as a scaffold for this process that could be observed in the microscopic analysis (Figure 3). A similar effect was observed by Borkowski et al. [37], who detected the increased interaction of cells with silica nanoparticles in the presence of positively charged ionic liquids. The role of silica in our studies can also be supported by the fact that we did not observe such a strong agglomeration effect in the additional samples that contained only TiO_2_. 

The morphology and viability provided additional information on the observed rapid agglomeration of cells. The addition of nanocomposite caused agglomeration that was dependent on the concentration of nanomaterial, which is shown in Figure 3 and Figure 4. Interestingly, the biochemical evaluation did not show a significant reduction in the viability of bacteria after the population growth. Additional alamarBlue^®^ assay, which was executed at the tenth hour of the experiment for establishing the kinetic growth, showed no significant differences (*P* < 0.05) between samples. This may demonstrate that even though bacteria were clustered together, their viability remained stable or their metabolic activity was upregulated, which could also be the case in the biofilm formation assays (Figure 5). This result is surprising in the light of data indicating the antibacterial activity of TiO_2_. However, it should be stressed that the antibacterial effect may be dependent on the particle size, the concentration of nanomaterial, its crystal phase, and the mode of activation [35,38]. In the used nanotubes, TiO_2_ accounted for 35–45% of the composite. Nevertheless, in the highest concentration, there was enough nanomaterial to show antibacterial activity [38]. Furthermore, it was shown by Horst et al. [30] that *P. aeruginosa* can dissolute agglomerated TiO_2_ nanoparticles through adsorption on the cell surface. It was shown that microorganisms can interact with these substances and possibly absorb the nanoparticles, which we have previously observed on a *Streptomyces* model [25]. 

Exopolymeric substances that are produced by biofilming cells can additionally increase the adsorption of this nanomaterial from water [39]. Biofilm formation assays conducted in our studies provided some insights into the dynamics of bacterial biofilm formation during the exposure to titanium dioxide/silica nanotubes. Similar to the results shown by Maurer-Jones et al. [24], we also observed that the photocatalytic nanomaterial may affect the biofilm formation and physiology of bacteria. Moreover, Ouyang et al. [40] showed that, on ZnO nanoparticles, sublethal concentrations may stimulate bacteria toward the secretion of biofilm-related signaling molecules. However, in our study, the biomass of biofilms was not affected in the majority of cases, while its respiration was highly upregulated after 48 h. The comparison to the control samples indicated that the presence of the nanomaterials in the growth environment could contribute to the aggregation of cells, although the material itself was not agglomerating on the surface of the polystyrene plate.

The gene expression study indicated the slight upregulation of the *mexA* gene, which encodes the efflux pump responsible for the removal of substances from cells. At this stage, it is unclear whether the effect was caused by a direct interaction between cells and nanoparticles or the agglomeration of cells. However, based on our previous studies, we hypothesize that this effect could be caused by TiO_2_ nanoparticles that detached from a silica shell and that were interacting with cells, possibly being absorbed. A similar effect was observed in previous studies on *Escherichia coli* [15] and *Streptomyces* sp. [25]. In the second case, the EDX analysis on TEM revealed a signal coming from titanium inside *pseudomycelium*, in regions containing deposits of polyphosphate. Interestingly, other authors have shown that the exposition to QD may lead to the overexpression of antibiotic resistance-related genes, as was observed in PAO1. The transcriptomic approach revealed that sublethal QD exposure upregulates global stress defense mechanisms in *P. aeruginosa* PAO1. There are two main mechanisms of a cell’s defense against heavy metal toxicity: the increased expression of efflux pumps and the overproduction of antioxidant enzymes [29]. These findings suggest that, in our case, the nanomaterial could similarly stimulate the used strain; however, the effect (i.e., suspected increased antibiotic resistance) was not observable on agar plates. Therefore, further investigation is necessary to describe the mechanism responsible for the observed phenomenon. 

The enhanced inhibition of the biofilm formation (in 72-hour-old cultures) in the case of the highest concentration of nanocomposite used can be explained by the cell/nanocomposite ratio that resulted in higher doses of mt-SiO_2_/TiO_2_ per cell. A high cell/nanocomposite ratio could result in the death of the part of the initially seeded bacteria, increasing the number of the formed microcolonies that were gathering around the nanomaterial. This process probably caused the characteristic peak in the OD of the samples between the second and fourth hours of incubation (Figure 4). In principle, lower NP concentration can cause some cell damage, poses a high probability of cell recovery, and, as a result, causes the acceleration of biofilm development. As indicated above, such results were observed for ZnO nanoparticles, where lower concentrations accelerated biofilm formation due to the cell response. On the other hand, higher concentrations decreased the biomass formation and the number of biofilm formation centers [40,41]. In the current study, the measured biomass of biofilms did not generally differ from the control samples. 

Additionally, the overproduction of antioxidant enzymes and the increased expression of efflux pumps due to heavy metal (HM)-related stress could increase the metabolic activity of cells [29] that was observed for 48-hour biofilms (Figure 5). Several genes were identified to play an important role in the heavy metal response and were also induced by HM-shocked cultures (superoxide dismutase (*sodM*), catalase (*katB*), genes involved in organic hydroperoxide resistance (*ohr* and *ahpF*), and genes involved in the DNA SOS response/DNA damage and repair (*dinP*, *recA*, and *recN*) [42,43,44]. The active efflux pump plays an important role in heavy metal tolerance [42], thus the (slight) upregulation of the *mexA* gene confirms that cells might try to push the smaller TiO_2_ nanoparticles or molecules out of the cells. 

These results create ground for further studies on the stimulation of bacteria with nanomaterials. The next focus should be on mechanisms that govern the phenomena observed in current study. Our findings also confirm the importance of using *P. aeruginosa* as a model for studying the response to nanomaterials in terms of bacterial physiology and metabolism instead of commonly used bacteria such as *E. coli* [45].

## 4. Materials and Methods

### 4.1. Materials

*P. aeruginosa* PAO1, a Gram-negative, motile, and biofilm-forming reference bacterium, served as the biological material for all experiments. The microorganism was selected based on its known physiological and adaptational properties [29,46].

The nanomaterial used for this study was comprised of mesoporous silica nanotubes functionalized with titanium dioxide (mt-SiO_2_/TiO_2_). Carbon nanotubes that were used for the synthesis of the mesoporous shell were purchased from Shenzhen Nanotech Port Co. (Shenzhen, China). Hexadecyl(trimethyl)ammonium bromide (CTAB), silica (tetraethyl orthosilicate—TEOS), and titanium dioxide precursor (titanium (IV) butoxide—TBT) was purchased from Sigma-Aldrich. Ethanol, ammonium, and n-propanol were provided by Chempure (Piekary Slaskie, Poland) and Avantor Performance Materials Poland (Gliwice, Poland). The nanocomposite was used in three working concentrations: 0.1, 0.05, and 0.01 mg/mL. In previous studies, lower concentrations were found to have inhibitory effects against *E. coli* [15], although they were below the minimal inhibitory concentration for *P. aeruginosa* PAO1 [47].

### 4.2. Synthesis of Titanium Dioxide/Silica Nanotubes (mt-SiO_2_/TiO_2_)

The synthesis and functionalization of mesoporous silica nanotubes with titanium dioxide were previously described by [15,34]. For the preparation of mt-SiO_2_/TiO_2_, functionalized multiwalled carbon nanotubes (Shenzhen Nanotech Port Co., Shenzhen, China) were dispersed by ultrasonication in the solution containing 0.3 g CTAB, 1.0 g of NH_3_·H_2_O, 60 mL of ethanol (Chempure), and 80 mL of deionized water (H_2_O). When the mixture reached a homogeneous state, 0.4 mL of TEOS was added to the reaction and, subsequently, stirred at room temperature for 24 h. The final product was obtained through evaporation. Afterward, the CTAB was burned from the silica-carbon nanotubes in the air at 400 °C for two hours. The obtained mesoporous silica-carbon nanotubes were sonicated for three hours in 5 mL of concentrated tetrabutyl titanate (TBT; Merck, Darmstadt, Germany). Following the incubation, the TBT and silica-carbon nanomaterials were diluted with n-propanol and collected after centrifugation. Then, the sample was washed several times with n-propanol to remove an excess of TBT and dispersed in ethanol to hydrolyze the titanium dioxide precursor. The mesoporous silica nanotubes with titanium dioxide were calcined at 600 °C for four hours to remove the CNT core and transform the amorphous titanium dioxide to the anatase phase.

The aqueous solution of titanium dioxide/silica nanotubes suspended in ultrapure water was used in three final concentrations of 0.1, 0.05, and 0.01 mg/mL. The nanomaterial was exposed to the UVC light for five minutes prior to being used in experiments.

### 4.3. Preparation of Bacteria

*P. aeruginosa* PAO1 was obtained from the collection of the Institute of Microbiology at the University of Warsaw and was stored in the collection of the Department of Microbiology and Biotechnology at the West Pomeranian University of Technology, Szczecin (Poland). The bacteria were kept at −20 °C in a trypticase soy broth (TSB) medium containing 10% glycerol. Before the study, the microorganism was revived on trypticase soy agar (TSA) and incubated at 30 °C for 24 h. For experiments that required the use of liquid culture, bacteria were inoculated to falcon tubes (15 mL) containing 10 mL of TSB and incubated overnight at 30 °C on a rotary shaker.

### 4.4. Viability Testing and Observation of Cells Agglomeration

The morphology of colonies was analyzed on a TSA medium after 24 h growth at 30 °C following contact with mt-SiO_2_/TiO_2_ nanotubes. Bacteria were streaked to a fresh medium with a 1µL inoculation loop at every stage of the experiments as described below.

The morphology of cells was analyzed with phase-contrast microscopy on a Delta L-1000 microscope (Delta optical, Nowe Osiny, Poland) in 10 μL droplets covered with a cover slide. Cells were harvested from cultures with a 1 μL inoculation loop and transferred to 100 μL of sterile PBS. Afterward, 1 μL of nanomaterial suspension was added on the microscope slide to 9 μL of bacterial suspension so that the final concentration of nanomaterial was equal to 0.1 mg/mL, 0.05 mg/mL, or 0.01 mg/mL. An additional control containing only TiO_2_ was also included.

The growth curves were determined by the observation of behavior and measurements of the OD of cultures at the wavelength 600 nm. For this purpose, bacteria were cultured in 20 mL of TSB medium in small 100 mL flasks at 30 °C for ten hours. Bacteria were also cultured in 24-well polystyrene plates to compare results and observations between experimental settings. In each method, measurements were taken every 30 min in triplicate. At the end of incubation, the metabolic activity of cultures was measured using alamarBlue^®^, according to the user’s manual. During the assay, samples were incubated at 30 °C for 60 min. Viability of *P. aeruginosa* PAO1 at the endpoint of growth kinetics studies was performed with the alamarBlue^®^ assay. Fluorescence (λ_ex_ = 520 nm, λ_em_ = 590 nm) was measured on BioTek Synergy H1 spectrophotometer (Winooski, VT, USA). Measurements were conducted according to the user’s manual. Every experiment was conducted in triplicate, while every sample was carried out in six replicates. The results were compared to the control samples without bacteria but containing nanomaterial and bacteriological medium alone. Additionally, alamarBlue^®^ assay was tested for interference with nanomaterial to ensure that obtained data were not impaired.

Additionally, flow cytometry was applied to investigate the influence of the nanocomposite on the scale of the bacterial population. Experiments were performed with a Cube 8 (Sysmex, Kobe, Japan) flow cytometer equipped with a blue laser (488 nm). Forward scatter (FSC) and side scatter (SSC) detectors were used for analysis. Experiments were conducted under the following settings: threshold set on 100, FSC voltage—200 V, and SSC voltage—250 V. The quantity of each sample was set to 10 μL. The flow was set to 0.5 μL/s. Each analysis contained background controls with PBS, PBS with bacteriological medium, PBS with nanomaterials, and PBS with bacteriological medium and nanomaterials.

### 4.5. Biofilm Viability and Biomass

Tests were conducted at three different times: 24, 48 and 72 h at 37 °C to examine various stages of biofilm formation. Biofilms were cultured with and without shaking to compare gained results. In the first stage, biofilms were washed five times with sterile PBS. Afterward, TSB containing 10% alamarBlue^®^ (1 mg/mL) was added to wells, and plates were agitated for 3 s and incubated at 30 °C for 45 min. Then, fluorescence (λ_ex_ = 520 nm, λ_em_ = 590 nm) was recorded on BioTek Synergy H1 spectrophotometer (Winooski, VT, USA). The relative biomass of biofilms was examined by a crystal violet staining assay on 96-well titration plates according to Merritt et al. [48]. All samples were measured in six repetitions.

### 4.6. Gene Expression Studies

Bacteria were cultured with nanomaterial as described above. In the mid-exponential growth phase, 1 mL of cultures was transferred to 1.5 mL tubes for RNA extraction. Isolation of genetic material was conducted by the use of a Genomic RNA mini kit (A&A Biotechnology, Gdynia, Poland), according to the user’s manual. Reverse transcription was conducted with the Superscript IV VILO Master Mix (ThermoFisher Scientific, Waltham, MA, USA), according to the user’s manual. The quantity of gained material was measured with Qubit 2.0 fluorometer. The amount of cDNA was unified between samples with PCR-grade water. 

Six genes were taken into consideration in the gene expression studies. After *recA* in the qPCR reaction. Primers for the reaction are shown in Table 2. Conditions used for the reaction were previously described by Savli et al. [49]. Experiments were conducted on the Lightcycler 96 (Roche, Basel, Switzerland) thermocycler. Each amplification was followed by an HRM step to confirm the specificity of the reaction. All samples were studied in triplicate, while gained tendencies were verified in three separate experiments. 

### 4.7. Antibiotic Susceptibility on Media Containing Activated Nanomaterials

The nanomaterial suspended in ultrapure water (1 mg/mL) was sonicated for 30 min and vortexed before use. There was 180 mL of Muller-Hinton Agar medium prepared and aseptically portioned into two batches: one tested batch with the addition of 10 mL of nanomaterial suspension, and one control batch with the addition of 10 mL of distilled water. Before the experiment, the media was exposed to UV light for five minutes. The susceptibility to five antibiotics was determined using the Kirby-Bauer disk diffusion method, carried out according to EUCAST recommendations (Version 9.0, 2019, for gentamycin and Version 10.0, 2020, for the rest of tested antimicrobials). The group of tested antibiotics included aztreonam (ATM, 30 μg), cefepime (FEP, 30 μg), ciprofloxacin (CIP, 5 μg), gentamicin (CN, 10 μg), and meropenem (MEM, 10 μg). The size of the zones of growth inhibition allowed the classification of the strain as R (resistant) or S (susceptible). 

### 4.8. Statistical Analysis

The results were analyzed with one-way ANOVA and calculated with Statistica 13.1 software (Statsoft, Krakow, Poland). Post hoc testing included Tukey’s test. Assumptions were also tested. Every quantitative experiment was conducted in triplicate with six repetitions of each studied variable in each experiment. Results with *P* < 0.05 were considered significant. Standard deviation (SD) was used as error bars in Figure 4, Figure 5 and Figure 6. 

## 5. Conclusions

Titanium dioxide/silica nanotubes induce agglomeration in *P. aeruginosa* PAO1, which may be associated with the oxidative stress induced by the presence of TiO_2_ nanoparticles. This process is highly upregulated when the nanocomposite is previously irradiated with UV light. Although the mobility of cells decreased in samples containing nanocomposite, the attached biofilm biomass was not significantly increased and in higher concentrations was even decreased. Despite this finding, the respiration was upregulated, particularly in 48-hour biofilms. Cells were gathering around the nanocomposite until it was completely covered, allowing the remaining bacteria to spread the population. Comparable viability between samples suggests that agglomerated cells maintain their activity or the respiration is upregulated, which is to be determined in further studies. Such changes in the physiology of *P. aeruginosa* PAO1 along with upregulation in the expression of gene coding an efflux pump indicate that using these nanomaterials in therapy may potentially induce the virulence of this bacterium. On the other hand, the observed phenomena may find application in biotechnological processes, where induction of respiration could be used to optimize biomass production. The agglomeration could be used to harvest biomass from the bioreactors.

## Figures and Tables

**Table 1:** Antibiotic susceptibility on treated and control media.

Antibiotic	Control (mm)	SD	Treated (mm)	SD	EUCAST *
CN	21.00	±0.00	19.33	±0.58	15
FEP	30.67	±0.58	29.67	±0.58	21
ATM	28.67	±0.58	29.00	±0.00	18
MEM	36.50	±0.71	35.00	±0.00	18
CIP	35.00	±1.00	36.00	±0.00	26

* EUCAST values indicating resistance to the antibiotic.

**Table 2:** Genes and primers that were used in this study.

Primer Name	Gene Function	Sequence (5′–3′)	Reference
ampC-f	Chromosomal beta lactamase	AGATTCCCCTGCCTGTGC	[49]
ampC-r		GGCGGTGAAGGTCTTGCT	
recA-f	Recombinase A	TCCGCAGGTAGCACTCAGTTC	[50]
recA-r		AAGCCGGATTCATAGGTGGTG	
oprD-f	Outer membrane protein	ATCTACCGCACAAACGATGAG	[51]
oprD-r		GCCGAAGCCGATATAATCAAACG	
oprL-f	Peptidoglycan-associated lipoprotein	ATGGAAATGCTGAAATTCGGC	[52]
oprL-r		CTTCTTCAGCTCGACGCGACG	
gyrA-f	Gyrase	TGTGCTTTATGCCATGAGCGA	[53]
gyrA-r		TCCACCGAACCGAAGTTGC	
mexA-f	Efflux pump	CTCGACCCGATCTACGTC	[51]
mexA-r		GTCTTCACCTCGACACCC	

## References

[1] Branski L.K., Al-Mousawi A., Rivero H., Jeschke M.G., Sanford A.P., Herndon D.N. (2009). Emerging Infections in Burns. Surg. Infect. (Larchmt).

[2] Winstanley C., O’Brien S., Brockhurst M.A. (2016). Pseudomonas aeruginosa Evolutionary Adaptation and Diversification in Cystic Fibrosis Chronic Lung Infections. Trends Microbiol..

[3] Newman J.W., Floyd R.V., Fothergill J.L. (2017). The contribution of Pseudomonas aeruginosa virulence factors and host factors in the establishment of urinary tract infections. FEMS Microbiol. Lett..

[4] Rosenthal V.D., Al-Abdely H.M., Ali El-Kholy A., Aziz AlKhawaja S.A., Leblebicioglu H., Mehta Y., Rai V., Viet Hung N., Sami Kanj S., Foda Salama M. (2016). International Nosocomial Infection Control Consortium report, data summary of 50 countries for 2010–2015: Device-associated module. Am. J. Infect. Control..

[5] Wojciuk B., Salabura A., Grygorcewicz B., Kędzierska K., Ciechanowski K., Dołęgowska B. (2019). Urobiome: In Sickness and in Health. Microorganisms.

[6] Lotfabad T.B., Shahcheraghi F., Shooraj F. (2013). Assessment of antibacterial capability of rhamnolipids produced by two indigenous Pseudomonas aeruginosa strains. Jundishapur J. Microbiol..

[7] Panghal M., Singh K., Kadyan S., Chaudary U., Yadav J.P. (2015). The analysis of distribution of multidrug resistant Pseudomonas and Bacillus species from burn patients and burn ward environment. Burns.

[8] Ding C., Yang Z., Wang J., Liu X., Cao Y., Pan Y., Han L., Zhan S. (2016). Prevalence of Pseudomonas aeruginosa and antimicrobial-resistant Pseudomonas aeruginosa in patients with pneumonia in mainland China: A systematic review and meta-analysis. Int. J. Infect. Dis..

[9] WHO Global Priority List of Antibiotic-Resistant Bacteria to Guide Research, Discovery, and Development of New Antibiotics. www.who.int/medicines/publications/global-priority-list-antibiotic-resistant-bacteria/en/.

[10] Sikora P., Cendrowski K., Markowska-Szczupak A., Horszczaruk E., Mijowska E. (2017). The effects of silica/titania nanocomposite on the mechanical and bactericidal properties of cement mortars. Constr. Build. Mater..

[11] Sikora P., Augustyniak A., Cendrowski K., Horszczaruk E., Rucinska T., Nawrotek P., Mijowska E. (2016). Characterization of Mechanical and Bactericidal Properties of Cement Mortars Containing Waste Glass Aggregate and Nanomaterials. Materials (Basel).

[12] Iavicoli I., Leso V., Beezhold D.H., Shvedova A.A. (2017). Nanotechnology in agriculture: Opportunities, toxicological implications, and occupational risks. Toxicol. Appl. Pharmacol..

[13] Trukawka M., Cendrowski K., Peruzynska M., Augustyniak A., Nawrotek P., Drozdzik M., Mijowska E. (2019). Carbonized metal–organic frameworks with trapped cobalt nanoparticles as biocompatible and efficient azo-dye adsorbent. Environ. Sci. Eur..

[14] Nawrotek P., Augustyniak A. (2015). Nanotechnology in microbiology—Selected aspects | Nanotechnologia w mikrobiologii—wybrane aspekty. Postep. Mikrobiol..

[15] Cendrowski K., Peruzynska M., Markowska-Szczupak A., Chen X., Wajda A., Lapczuk J., Kurzawski M., Kalenczuk R.J., Drozdzik M., Mijowska E. (2014). Antibacterial performance of nanocrystallined titania confined in mesoporous silica nanotubes. Biomed. Microdevices..

[16] Borkowski A., Cłapa T., Szala M., Gąsiński A., Selwet M. (2016). Synthesis of SiC/Ag/Cellulose Nanocomposite and Its Antibacterial Activity by Reactive Oxygen Species Generation. Nanomaterials.

[17] Piszczek P., Lewandowska Ż., Radtke A., Jędrzejewski T., Kozak W., Sadowska B., Szubka M., Talik E., Fiori F. (2017). Biocompatibility of Titania Nanotube Coatings Enriched with Silver Nanograins by Chemical Vapor Deposition. Nanomaterials.

[18] Li Q., Mahendra S., Lyon D.Y., Brunet L., Liga M.V., Li D., Alvarez P.J.J. (2008). Antimicrobial nanomaterials for water disinfection and microbial control: Potential applications and implications. Water Res..

[19] Holden P., Schimel J.P., Godwin H. (2014). Five reasons to use bacteria when assessing manufactured nanomaterial environmental hazards and fates. Curr. Opin. Biotechnol..

[20] Xu W., Xie W., Huang X., Chen X., Huang N., Wang X., Liu J. (2017). The graphene oxide and chitosan biopolymer loads TiO_2_ for antibacterial and preservative research. Food Chem..

[21] Lemire J., Harrison J.J., Turner R.J. (2013). Antimicrobial activity of metals: Mechanisms, molecular targets and applications. Nat. Rev. Microbiol..

[22] Ge Y., Schimel J.P., Holden P. (2011). a Evidence for negative effects of TiO2 and ZnO nanoparticles on soil bacterial communities. Environ. Sci. Technol..

[23] Ge Y., Schimel J.P., Holdena P. (2012). Identification of soil bacteria susceptible to TiO_2_ and ZnO nanoparticles. Appl. Environ. Microbiol..

[24] Maurer-Jones M.A., Gunsolus I.L., Meyer B.M., Christenson C.J., Haynes C.L. (2013). Impact of TiO_2_ nanoparticles on growth, biofilm formation, and flavin secretion in Shewanella oneidensis. Anal. Chem..

[25] Augustyniak A., Cendrowski K., Nawrotek P., Barylak M., Mijowska E. (2016). Investigating the Interaction Between Streptomyces sp. and Titania/Silica Nanospheres. Water. Air. Soil Pollut..

[26] Yong Y.-C., Wu X.-Y., Sun J.-Z., Cao Y.-X., Song H. (2015). Engineering quorum sensing signaling of Pseudomonas for enhanced wastewater treatment and electricity harvest: A review. Chemosphere.

[27] Hwang I.Y., Koh E., Wong A., March J.C., Bentley W.E., Lee Y.S., Chang M.W. (2017). Engineered probiotic Escherichia coli can eliminate and prevent Pseudomonas aeruginosa gut infection in animal models. Nat. Commun..

[28] Mccaughey L.C., Josts I., Grinter R., White P., Byron O., Tucker N.P., Matthews J.M., Kleanthous C., Whitchurch C.B., Walker D. (2016). Discovery, characterization and in vivo activity of pyocin SD2, a protein antibiotic from Pseudomonas aeruginosa. Biochem. J..

[29] Yang Y., Mathieu J.M., Chattopadhyay S., Miller J.T., Wu T., Shibata T., Guo W., Alvarez P.J.J. (2012). Defense mechanisms of pseudomonas aeruginosa pao1 against quantum dots and their released heavy metals. ACS Nano.

[30] Horst A.M., Neal A.C., Mielke R.E., Sislian P.R., Suh W.H., Mädler L., Stucky G.D., Holden P. (2010). Dispersion of TiO_2_ nanoparticle agglomerates by Pseudomonas aeruginosa. Appl. Environ. Microbiol..

[31] Shivaji S., Madhu S., Singh S. (2011). Extracellular synthesis of antibacterial silver nanoparticles using psychrophilic bacteria. Process Biochem..

[32] Husseiny M.I., El-Aziz M.A., Badr Y., Mahmoud M.A. (2007). Biosynthesis of gold nanoparticles using Pseudomonas aeruginosa. Spectrochim. Acta Part A Mol. Biomol. Spectrosc..

[33] Farias C.B.B., Silva A.F., Rufino R.D., Luna J.M., Souza J.E.G., Sarubbo L.A. (2014). Synthesis of silver nanoparticles using a biosurfactant produced in low-cost medium as stabilizing agent. Electron. J. Biotechnol..

[34] Cendrowski K. (2018). Titania/mesoporous silica nanotubes with efficient photocatalytic properties. Polish, J. Chem. Technol..

[35] Verdier T., Bertron A., Erable B., Roques C. (2018). Bacterial Biofilm Characterization and Microscopic Evaluation of the Antibacterial Properties of a Photocatalytic Coating Protecting Building Material. Coatings.

[36] Baptista P.V., McCusker M.P., Carvalho A., Ferreira D.A., Mohan N.M., Martins M., Fernandes A.R. (2018). Nano-strategies to fight multidrug resistant bacteria-”A Battle of the Titans”. Front. Microbiol..

[37] Borkowski A., Syczewski M., Czarnecka-Skwarek A. (2019). Ionic liquids strongly affect the interaction of bacteria with magnesium oxide and silica nanoparticles. RSC Adv..

[38] Lin X., Li J., Ma S., Liu G., Yang K., Tong M., Lin D. (2014). Toxicity of TiO2 Nanoparticles to Escherichia coli: Effects of Particle Size, Crystal Phase and Water Chemistry. PLoS ONE.

[39] Gupta G.S., Kumar A., Shanker R., Dhawan A. (2016). Assessment of agglomeration, co-sedimentation and trophic transfer of titanium dioxide nanoparticles in a laboratory-scale predator-prey model system. Sci. Rep..

[40] Ouyang K., Mortimer M., Holden P., Cai P., Wu Y., Gao C., Huang Q. (2020). Towards a better understanding of Pseudomonas putida biofilm formation in the presence of ZnO nanoparticles (NPs): Role of NP concentration. Environ. Int..

[41] Capdevila D.A., Wang J., Giedroc D.P. (2016). Bacterial strategies to maintain zinc metallostasis at the host-pathogen interface. J. Biol. Chem..

[42] Teitzel G.M., Geddie A., De Long S.K., Jo Kirisits M., Whiteley M., Parsek M.R., Cu H., Cu H. (2006). Survival and Growth in the Presence of Elevated Copper: Transcriptional Profiling of Copper-Stressed Pseudomonas aeruginosa. J. Bacteriol..

[43] Salunkhe P., Töpfer T., Buer J., Tümmler B. (2005). Genome-Wide Transcriptional Pro ling of the Steady-State Response of Pseudomonas aeruginosa to Hydrogen Peroxide. J. Bacteriol..

[44] Palma M., DeLuca D., Worgall S., Quadri L.E.N. (2004). Transcriptome Analysis of the Response of Pseudomonas aeruginosa to Hydrogen Peroxide. J. Bacteriol..

[45] Sikora P., Augustyniak A., Cendrowski K., Nawrotek P., Mijowska E. (2018). Antimicrobial Activity of Al_2_O_3_, CuO, Fe_3_O_4_, and ZnO Nanoparticles in Scope of Their Further Application in Cement-Based Building Materials. Nanomaterials.

[46] Palleroni N.J. (2015). Pseudomonas.

[47] Augustyniak A. (2019). The Influence of Nanomaterials on the Environmental Bacteria of Potential Biotechnological Significance. Ph.D. Thesis.

[48] Merritt J.H., Kadouri D.E., O’Toole G.A. (2015). Growing and Analyzing Static Biofilms. Curr. Protoc. Microbiol..

[49] Savli H., Karadenizli A., Kolayli F., Gundes S., Ozbek U., Vahaboglu H. (2003). Expression stability of six housekeeping genes: A proposal for resistance gene quantification studies of Pseudomonas aeruginosa by real-time quantitative RT-PCR. J. Med. Microbiol..

[50] Alqarni B., Colley B., Klebensberger J., McDougald D., Rice S.A. (2016). Expression stability of 13 housekeeping genes during carbon starvation of Pseudomonas aeruginosa. J. Microbiol. Methods.

[51] Arabestani M.R., Rajabpour M., Mashouf R.Y., Alikhani M.Y., Mousavi S.M. (2014). Expression of efflux pump mexab-oprm and oprd of pseudomonas aeruginosa strains isolated from clinical samples using qrt-pcr. Arch. Iran. Med..

[52] Aedekerk S., Diggle S.P., Song Z., Høiby N., Cornelis P., Williams P., Cámara M. (2005). The MexGHI-OpmD multidrug efflux pump controls growth, antibiotic susceptibility and virulence in Pseudomonas aeruginosa via 4-quinolone-dependent cell-to-cell communication. Microbiology.

[53] Bragonzi A., Worlitzsch D., Pier G.B., Timpert P., Ulrich M., Hentzer M., Andersen J.B., Givskov M., Conese M., Döring G. (2005). Nonmucoid Pseudomonas aeruginosa Expresses Alginate in the Lungs of Patients with Cystic Fibrosis and in a Mouse Model. J. Infect. Dis..

